# Comparison of Prognostic Performance between Procalcitonin and Procalcitonin-to-Albumin Ratio in Post Cardiac Arrest Syndrome

**DOI:** 10.3390/jcm12144568

**Published:** 2023-07-09

**Authors:** Ju Hee Yoon, Woo Sung Choi, Yong Su Lim, Jae Ho Jang

**Affiliations:** 1 Department of Emergency Medicine, Gachon University Gil Medical Center, Incheon 21565, Republic of Korea; yjh920722@gilhospital.com; 2 Department of Emergency Medicine, Gachon University College of Medicine, Incheon 21565, Republic of Korea; yongem@gilhospitl.com (Y.S.L.); jhjang@gilhospital.com (J.H.J.)

**Keywords:** post cardiac arrest syndrome, prognosis, procalcitonin to albumin ratio, mortality, neurologic outcome

## Abstract

(1) Background: Post-cardiac arrest syndrome (PCAS) is a type of global ischemic reperfusion injury that occurs after the return of spontaneous circulation (ROSC). The procalcitonin to albumin ratio (PAR) has been studied as an independent prognostic factor of various diseases. There are no previous studies of PAR in patients with PCAS. We assessed if PAR is more effective than procalcitonin (PCT) in predicting prognosis for patients with PCAS. (2) Methods: This retrospective cohort study included a total of 187 patients with PCAS after non-traumatic out-of-hospital cardiac arrest (OHCA) between January 2016 and December 2020. Multivariate logistic regression analysis was conducted to assess the association between PAR and PCAS prognosis. The predictive performance of PAR was compared with PCT via the receiver-operating characteristic (ROC) analysis and DeLong test.; (3) Results: PAR at 24 and 48 h after hospital admission were independently associated with one-month neurological outcome (OR: 1.167, 95% CI: 1.023–1.330; OR: 1.077, 95% CI: 1.012–1.146, *p* < 0.05). By ROC analysis, PAR showed better performance over PCT at 48 h after admission in predicting one-month CPC (0.763 vs. 0.772, *p* = 0.010). (4) Conclusions: Our findings suggest that PAR at 48 h after admission is more effective in predicting a one-month neurological outcome than PCT at 48 h after admission in patients with PCAS after OHCA.

## 1. Introduction

Postcardiac arrest syndrome (PCAS) results in high morbidity and mortality due to a series of life-threatening complications such as multiorgan failure and neurological damage [1,2,3]. PCAS is a type of global ischemic reperfusion injury that occurs after the return of spontaneous circulation (ROSC) by a mechanism similar to one in systemic inflammatory response syndrome (SIRS) or sepsis [4,5]. PCAS manifests various clinical courses leaving a significant impact on a patient’s long-term prognosis [2]. Thus, it is crucial to make precise and timely predictions about prognosis. Although several studies assessed prognostic factors, such as clinical circumstances, biomarkers, neuroimaging, and patient characteristics, in cardiac arrest patients, early prognostication remains challenging [2,6].

Blood biomarkers are useful predictors because they can be easily sampled at specific time points and can provide quantitative results. Since a systematic activation of inflammatory pathways occurs in PCAS, elevated inflammatory markers, such as white blood cell count (WBC), C-reactive protein (CRP) and procalcitonin (PCT), can be prognostic predictors in PCAS [2,7]. PCT, a prohormone of calcitonin consisting of 116 amino acids, is primarily produced during severe systemic inflammation resulting from bacterial infections. However, PCT is also closely related to the severity of systemic inflammation [8,9]. Recent studies have shown that PCT levels are strongly associated with patients with PCAS, and are used as prognostic indicators as well as specific indicators of infection in these patients [8,10,11]. An increase in PCT at an early stage of PCAS has been proven to be relevant in predicting neurological prognosis [10,12,13,14] and mortality [12,13,15,16]. Serum albumin (ALB), traditionally considered a marker of nutrition, have multiple vital functions in the body, such as protecting against free radicals, regulating fluid balance, preventing oxidative damage, supporting blood vessels, and reducing inflammation [17,18,19]. For these reasons, previous studies suggest that lower ALB level is associated with the severity and mortality of a wide variety of diseases [17,18,19]. Furthermore, ALB comprises approximately 80% of plasma colloid oncotic pressure [20]. PCAS itself increases vascular permeability as a result of a sepsis-like mechanism and consequently leads to hypoalbuminemia that can decrease intravascular volume and lead to inadequate blood flow to the vital organs [19,21,22].

Recently, a combination of ALB and indicators of systemic inflammation, such as CRP to ALB ratio (CAR), PCT to ALB ratio (PAR), and lactate to ALB ratio (LAR), has been extensively explored as an independent predictor of survival rate of various diseases [17,21,23,24,25,26,27]. The PAR in other diseases such as intracranial hemorrhage, urinary tract infection, sepsis, and COVID-19 infection has been reported as a reliable new indicator for predicting prognosis and severity [17,25,26,28]. However, there have been no studies that investigated the prognostic value of PAR in PCAS patients to the best of our knowledge.

Hence, we aimed to evaluate the prognostic value of PAR, in comparison with PCT, for the prediction of mortality and neurologic prognosis in patients with PCAS after non-traumatic out-of-hospital cardiac arrest (OHCA).

## 2. Materials and Methods

### 2.1. Study Population and Setting

This study is a single-center, retrospective cohort study at a tertiary university hospital located in a metropolitan city. Patients who successfully resuscitated from OHCA and received targeted temperature management (TTM) at 33~36 °C for 24 h between January 2016 and December 2020, were considered for this study. Exclusion criteria of the study were patients with (1) age below 18 years, (2) causes of CA, such as cerebrovascular accident and poisoning, which are independent risk factors for neurologic deterioration, (2) pre-existing severe neurological impairment prior to CA (Glasgow–Pittsburgh cerebral performance category, CPC ≥ 3), (3) end-stage non-cardiac disease before CA, and (4) time of death within 24 h after hospital admission. Hemodynamically unstable patients, who cannot tolerate hypothermia and were relatively contraindicated for TTM, underwent normothermic protocol. Therefore, our study only included patients who underwent TTM.

All advanced cardiovascular life support (ACLS) procedures were performed by emergency medicine physicians and paramedics.

This study was approved by the institutional review board of our hospital (GDIRB2023-156). Demographic data were obtained from a registry database of PCAS patients at our hospital based on the pre-and in-hospital medical records. All other data were gathered by retrospectively reviewing medical records.

### 2.2. Laboratory Measurements

We measured serum PCT and ALB immediately after admission to Emergency Room (at arrival), 24 h and 48 h after admission of all included patients. PAR was expressed as the ratio of PCT to ALB. Results at each time point were expressed as PCT_0_, PCT_24_, PCT_48_ PAR_0_, PAR_24_, PAR_48_, ALB_0_, ALB_24_, and ALB_48_. Initial arterial blood gas analysis for pH(pH_0)_ and basal lactate level (lactate_0_) were also analyzed. PCT values were measured using an enzyme-linked fluorescent immunoassay (bioMerieux VIDAS B.R.A.H.M.S. PCT, Craponne, France) on the Cobas e601 analyzer (Roche, Basel, Switzerland), and the detection limit was 0.05 ng/m.

### 2.3. Assessment of Clinical Outcomes

Our primary outcome is the evaluation of the superiority of PAR over PCT as a prognostic factor in patients with PCAS after non-traumatic OHCA, since PCT is widely accepted as an effective prognostic factor of mortality and neurologic outcome

We analyzed the one-month mortality rate (1m-mortality) and neurological outcomes (1m-CPC). In the 1m-mortality group, patients were subcategorized into survivor and non-survivor groups. If a patient expired at any point within 1 month, they were included in the non-survivor group. For neurologic evaluation, we used the Glasgow–Pittsburgh CPC scale (CPC, 1; Good cerebral performance, 2; Moderate cerebral disability, 3; Severe cerebral disability, 4; Coma or vegetative state, 5; Brain death). The patient’s basic CPC information was obtained through existing medical records and interviews with patients’ families. The CPC score after admission was measured daily by the attending physician if the patient is still hospitalized. The CPC score of discharged patients was measured by outpatient follow-up or telephone surveys for the purpose of follow-up over a certain period of time. In the 1m-CPC group, patients were divided into the good neurological outcome group (CPC 1 and CPC 2) and the poor neurological outcome group (CPC 3~5) for analysis.

### 2.4. Statistical Analysis

Data were analyzed using SPSS statistics for Windows, version 23.0 (IBM, SPSS Inc., Armonk, NY, USA) and MedCalc version 20.2 (MedCalc Inc., Mariakerke, Belgium). Continuous variables were presented as the median and interquartile range (IQR). Categorical variables were presented as numbers and percentages unless otherwise specified. Univariate analysis was performed using the Mann–Whitney U test for continuous variables and the chi-square test for categorical variables. Multivariable logistic regression analysis was performed to evaluate independent factors associated with 1m-mortality and 1m-CPC. Variables with *p*-values < 0.10 on univariate analysis were included in the multivariable regression model. Variables included in the multivariate logistic regression analysis were age, sex, the presence of bystander CPR and shockable rhythm, cardiac origin, and time from collapse to ROSC for 1m-mortality. Age, sex, shockable rhythm, cardiac origin, and time from collapse to ROSC were included for 1m-CPC. The predictive performance of PAR was compared with PCT via the receiver-operating characteristic (ROC) analysis. The AUC values at each time point were compared by the DeLong test. A *p*-value < 0.05 was considered statistically significant.

## 3. Results

### 3.1. Basal Characteristics of the Study Populations

**Figure 1:**
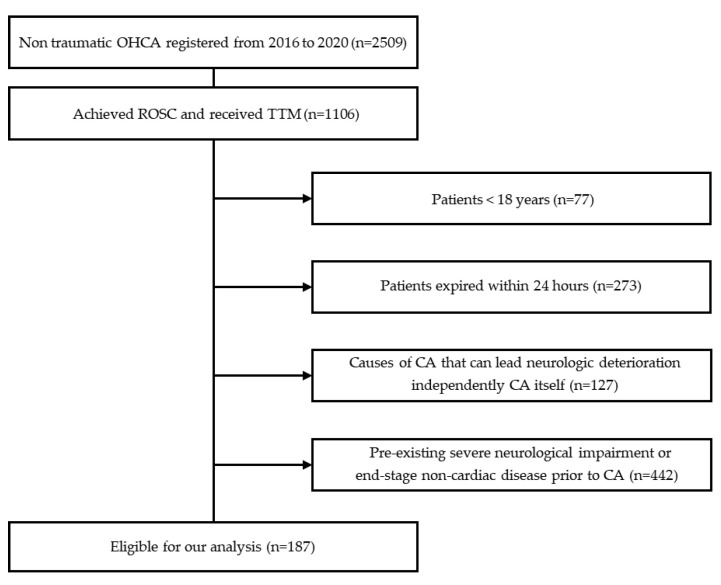
Schematic diagram showing the number of patients in the present study. OHCA, out-of-hospital cardiac arrest; CA, cardiac arrest.

During the observation period, 2509 patients were enrolled in our registry. Of those patients, 187 patients fulfilled all inclusion criteria and were enrolled in this study (Figure 1). The baseline characteristics of the patients are shown in Table 1.

Among 187 patients, 56 (29.9%) patients expired within 1 month. A total of 70 (37.4%) patients had good neurological outcomes (CPC 1–2), and 117 (62.6%) had poor neurological outcomes (CPC 3–5). Of the survivor group, 98 (52.4%) patients were male, and 49 (26.2%) patients in the good neurological outcome group were male. The median age of the survivor group was 55, while that of good neurological outcome was 56.

The non-survivor group had a higher incidence of cardiac arrest at a location other than the residence, lower incidence of bystander CPR, higher incidence of non-shockable rhythm, higher incidence of a non-cardiac cause of cardiac arrest, longer time to achieve ROSC, lower initial pH, and higher basal lactate levels (*p* < 0.05).

The poor neurological outcome group had a higher incidence of non-shockable rhythm, higher incidence of a non-cardiac cause of cardiac arrest, longer time to achieve ROSC, lower initial pH, and higher basal lactate levels (*p* < 0.05).

### 3.2. Comparison of PCT, ALB, and PAR According to 1m-mortality and 1m-CPC

**Figure 2:**
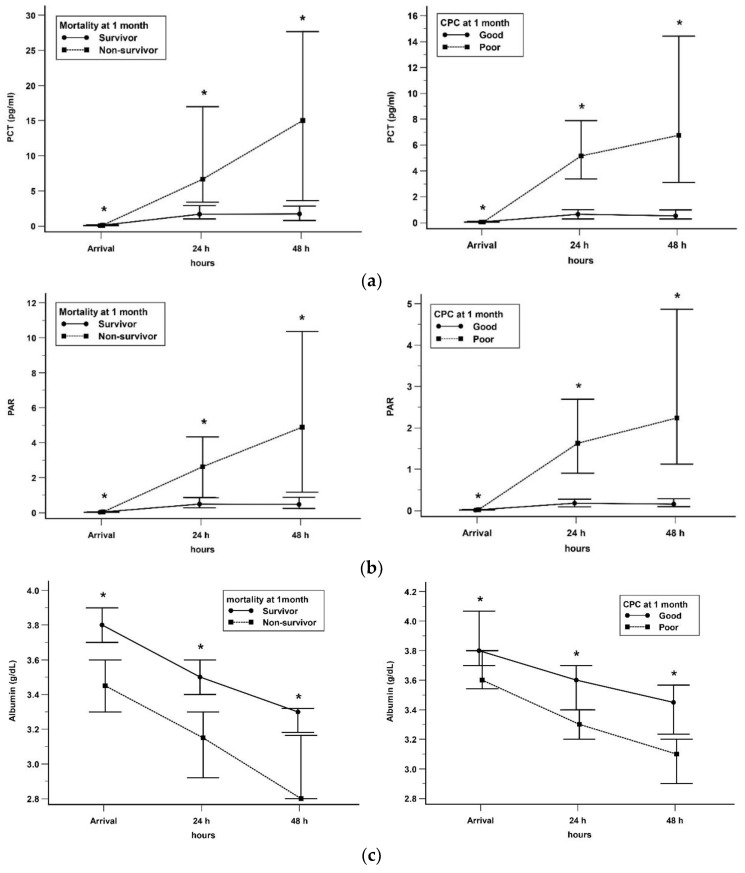
Serial change of biomarkers after hospital arrival according to 1m-mortality and neurological outcome. (**a**) PCT (**b**) PAR (**c**) ALB. Data are presented in median and 95% confidence interval. * mean statistically significant. PCT, procalcitonin; PAR, procalcitonin to albumin ratio.

The 1m-mortality and univariate analysis of PCT, ALB, and PAR were assessed according to the time elapse at admission, 24 h, and 48 h after admission. The laboratory data are presented in Table 2. PCT levels at all time points were higher in the non-survivor group than in the survivor group (*p* < 0.05). ALB levels at all time points were lower in the non-survivor group than in the survivor group (*p* < 0.05). The PAR levels at all time points of the survivor group were significantly lower than those of the non-survivor group (*p* < 0.05). The time course of each marker is presented in Figure 2. PCT and PAR levels in both groups showed an upward trend over time, whereas ALB levels showed a downward trend over time.

The 1m-CPC and univariate analysis of PCT, ALB, and PAR were also analyzed according to the time elapse at admission, 24 h, and 48 h after admission (Table 2). PCT levels were higher and ALB levels were lower in the poor CPC group than in the good CPC group at all time points (*p* < 0.05). PAR levels of the good CPC group were significantly lower than those of the poor CPC group at all time points (*p* < 0.05). The time course of each marker is presented in Figure 2. PCT and PAR levels in both groups increased over time, whereas ALB levels decreased over time.

### 3.3. Univariable and Multivariable Logistic Regression Analysis for Prediction of 1m-CPC and 1m-mortality

The associations between each variable and 1m-mortality is presented in Table 3. Lactate_0_ (odds ratio [OR]: 1.248, 95% confidence interval [CI]: 1.212–1.390) was positively associated with 1m-mortality, and pH_0_ (OR: 0.020, 95% CI: 0.002–0.181) was negatively associated with 1m-mortality (*p* < 0.05). PCT and PAR at all time points were not associated with 1m-mortality. The univariate and multivariate analyses were adjusted for age, sex, the presence of bystander CPR and shockable rhythm, cardiac origin, and time from collapse to ROSC.

The associations between each variable and 1m-CPC are presented in Table 4. PCT_24_ (OR: 1.055, 95% CI: 1.010-1.103), PCT_48_ (OR: 1.019, 95% CI: 1.001–1.037), PAR_24_ (OR: 1.167, 95% CI: 1.023–1.330) and PAR_48_ (OR: 1.077, 95% CI: 1.012–1.146) were independently shown positive associations with 1m-CPC (*p* < 0.05). pH_0_ (OR: 0.056, 95% CI: 0.007–0.475) was negatively associated with 1m-CPC (*p* < 0.05). The univariate analysis and multivariate analysis were adjusted for age, sex, shockable rhythm, cardiac origin, and time from collapse to ROSC.

### 3.4. ROC Analysis for Prediction of 1m-mortality and 1m-CPC

**Figure 3:**
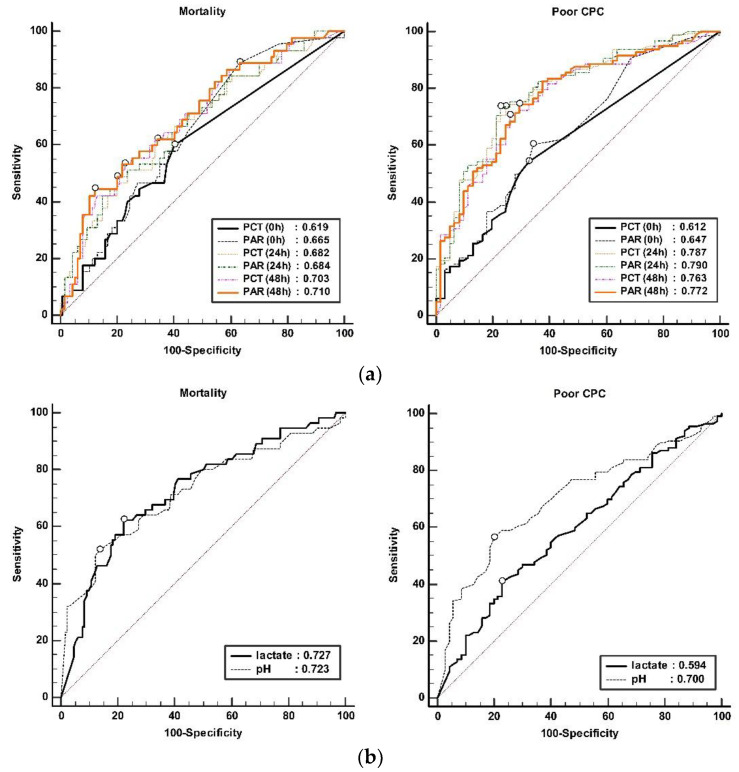
Comparison of ROC curve of (**a**) PCT and PAR at admission, 24 h, and 48 h after admission (**b**) Basal Lactate and pH for prediction of 1m-mortality and 1m-CPC. Box presented area under curve of each marker. PCT: procalcitonin; PAR: procalcitonin to albumin ratio.

As shown in Table 5, the AUCs of PCT_0_, PCT_24_, and PCT_48_ for predicting 1m-mortality in ROC analysis were 0.619, 0.682, and 0.703, respectively. Likewise, the AUCs of PAR_0,_ PAR_24_, and PAR_48_ were 0.665, 0.684, and 0.710, respectively. Only the AUC of PAR_0_ (*p* = 0.009) was higher than that of PCT_0_ for predicting 1m-mortality when comparing each time point. The AUC of PAR_24_ and PAR_48_ were not significantly different from the AUC of PCT_24_ and PCT_48_ (*p* = 0.587 and *p* = 0.052, respectively). The highest AUC value for the prediction of 1m-mortality was PAR_48_ (Figure 3). The AUCs of lactate and PH were 0.727 and 0.723, respectively, without statistical difference for prediction of 1m-mortality. These values are higher than the AUCs of PCT and PAR at all time points, but there was no statistical difference (lactate vs. PCT_0~48_: *p* = 0.071, 0.419, 0.441 for PCT; lactate vs. PAR_0~48_: *p* = 0.297, 0.442, 0.523; pH vs. PCT_0~48_: *p* = 0.104, 0.512, 0.464; pH vs. PAR_0~48_: *p* = 0.345, 0.544, 0.545) (Figure 3).

As shown in Table 6, the AUCs of PAR_0,_ PAR_24_, and PAR_48_ in ROC analysis for 1m-CPC prediction were 0.647, 0.790, and 0.772, respectively. The AUCs of PAR_0_ (0.612 vs. 0.647, *p* = 0.039) and PAR_48_ (0.763 vs. 0.772, *p* = 0.010) were significantly higher than that of PCT_0_ and PCT_48_ for predicting 1m-CPC. The AUC of PAR_24_ was not significantly different from the AUC of PCT_24_ (0.787 vs. 0.790, *p* = 0.494). The highest AUC value for the prediction of 1m-CPC was PAR_24_ (Figure 3). The AUCs of lactate and PH were 0.594 and 0.700, respectively, for the prediction of 1m-CPC. The AUC of lactate was significantly lower than the AUCs of PCT_24_, PCT_48_, PAR_24_, and PAR_48_ (lactate vs. PCT_0~48_: *p* = 0.833, <0.001, 0.005; lactate vs. PAR_0-48_: *p* = 0.405, <0.001, 0.003, respectively). The AUCs of pH were lower than those of PCT_24_, PCT_48_, PAR_24_, and PAR_48,_ but there was no statistical difference (pH vs. PCT_0~48_: *p* = 0.081, 0.197, 0.477; pH vs. PAR_0-48_: *p* = 0.310, 0.177, 0.375) (Figure 3).

## 4. Discussion

### 4.1. The Importance of Predicting the Prognosis of PCAS

The aim of the present study was to determine whether the PAR has higher efficacy for early prognostication over PCT in patients with PCAS after non-traumatic OHCA. To our knowledge, this study is the first one that investigates the association between PAR and the prognosis of patients with PCAS. The main finding of our study was PAR_48_ had higher predictability for the 1m-neurologic outcome than PCT_48_. PAR_24_ showed the highest performance based on AUC analysis; however, there was no statistical difference_._ There was neither association nor benefit of PAR at all times we investigated in predicting 1m-mortality.

PCAS consists of distinctive and intricate pathophysiological events: (1) brain damage after CA, (2) myocardial dysfunction following CA, and (3) global ischemia-reperfusion injury. Its mechanism is similar to the body’s reaction to SIRS or sepsis [2,4,5]. These reactions will vary in severity based on the degree of ischemic insult, the cause of cardiac arrest, and the patient’s basal health status.

It is important to make early predictions of prognosis with PCAS patients because PCAS progress through various clinical courses that affect a patient’s long-term prognosis significantly [2]. A proper clinical decision can identify a suitable population for aggressive intervention and avoid unnecessary interventions [29]. Although many previous studies have tried to assess more accurate prognostic factors [1,2,6,7,29,30,31,32], early prognostication remains challenging. It would be ideal to have a single accurate and efficient predictor, but no single reliable predictor has been identified, and there is a lack of studies in both quantity and quality [13]. Blood biomarkers gained attention due to their ease of sampling at specific time points, quantitative results, and independence from sedative effects during intensive care unit (ICU) care [15,29,33].

### 4.2. Serum ALB, PCT, PAR as a Prognostic Factor of PCAS

Serum ALB is the main protein in plasma, traditionally seen as a marker of nutrition, but now it is known to have multiple vital functions [17,18,19,34]. The protein protects against free radicals, regulates fluid balance, prevents oxidative damage, supports blood vessels, and reduces inflammation [17,18,19,34]. Furthermore, serum ALB comprises approximately 80% of plasma colloid oncotic pressure [20]. Hypoalbuminemia can decrease intravascular volume and lead to inadequate blood flow to the vital organs including the brain [19]. PCAS itself increases vascular permeability as a result of sepsis-like mechanism and consequently leads to the loss of serum ALB [21,22]. For these reasons, ALB has been considered an important prognostic factor. Several studies have demonstrated that low serum ALB levels are associated with unfavorable outcome of CA patients [34,35,36].

Nowadays, PCT, a soluble protein that is released into the bloodstream of patients with systemic inflammation [13], is widely accessible in the clinical setting. It increases particularly in bacterial infection; however, PCT levels may also rise considerably in patients who do not have sepsis, such as malignancies, organ transplantation, cardiogenic shock, and patients with PCAS [8,12,14,15,37,38,39,40]. Some previous studies [14,15] reported that PCT non-specifically increased in PCAS rather than early-onset infections. An increase in PCT at an early stage of PCAS, especially within 0–48 h, has been proven to be relevant in predicting neurological prognosis in patients with PCAS [10,12,13,14]. Based on these previous studies, we chose to analyze data at three specific time points: at admission, 24 h, and 48 h after admission. In neurologic outcome, PCT levels increased over time and our study suggests that PCT_24_ and PCT_48_ were significantly associated with 1m-CPC (OR: 1.055, 95% CI: 1.010–1.103; OR: 1.019, 95% CI: 1.001–1.037, *p* < 0.05). These results were concordant with previous results. Engel et al. reported that PCT over the first 24–48 h from admission was correlated with a 90-day neurological outcome [14]. Moreover, according to Jang et al., 24-h PCT levels were correlated with poor CPC scores after 3 months. As for mortality, there was no association between PCT and 1m-mortality. PCT levels showed an upward trend over time without statistical significance. This result was concordant with research by Engel et al., they found elevated PCT at day 1–2 after cardiac arrest showed a trend toward increased mortality but there was no statistical significance [14]. However, several previous studies contradict the results [13,15,16]. The differences may be explained by the following reasons: (1) The previous studies investigated the mortality within a relatively short term than our study (e.g., at 3 days, 14 days, or at discharge). (2) Moreover, we excluded patients with the end-stage disease before CA and patients who expired within 24 h after cardiac arrest, who showed extremely high PCT levels and have the potential to disturb the results.

A combination of ALB and indicators of systemic inflammation has been extensively studied as an independent factor that affects the disease severity and prognosis of patients with various diseases [17,21,23,24,25,26]. PAR is being studied as an efficient predictor in other severe diseases [17,25,26,28]. According to Lou et al., PAR was a helpful early diagnostic predictor for urosepsis and febrile UTI and had better prognostic value than other inflammatory biomarkers, such as CRP and leucocyte count [25]. As Tuba Et al. reported, PAR was related to COVID-19 disease severity and is a strong independent risk factor for ICU admission [28]. Wang et al. indicated that high PAR predicts progression to septic shock and mortality in patients with sepsis [26]. Despite such interests, there has been no study of PAR for PCAS prognostication. We aimed to assess PAR as a predictor of patients with PCAS after non-traumatic OHCA. By combining two factors, we hypothesized that the PAR would be a more reliable predictor than PCT. In univariate analysis, PCT levels in both the 1m-CPC group and 1m-mortality group showed an upward trend over time, whereas ALB levels showed a downward trend over time despite supplementation when ALB levels were below 3.0 g/dL. As a result, the PAR value showed a tendency to gradually increase over time, and it was expected that the reliability of the predictive value would increase as a combination of the two values. For mortality, the highest AUC value for prediction was PAR_48_, but there was no statistical difference with PCT_48_ (*p* = 0.052). Comparing each time point, the AUC of PAR_0_ was significantly higher than that of PCT_0_ (*p* = 0.009) for predicting 1m-mortality. However, as a result of multivariate analysis, there was no statistical significance of PAR for predicting 1m-mortality. For neurologic prognosis, PAR_24_, and PAR_48_ were independently associated with 1m-CPC (OR: 1.167, 95% CI: 1.023–1.330; OR: 1.077, 95% CI: 1.012–1.146, *p* < 0.05). The AUCs of PAR_0_ and PAR_48_ were significantly higher than that of PCT_0_ and PCT_48_ (*p* = 0.039, *p* = 0.010). PAR_24_ showed the highest AUC value for 1m-CPC numerically, but there was no statistically significant difference between the AUC of PAR_24_ and the AUC of PCT_24_ when compared with the DeLong test (*p* = 0.494). Furthermore, a lower pH level is considered a useful prognostic factor for neurologic outcome [41,42]; however, our results suggest that AUC values of pH_0_ were lower than those of PAR_24_ and PAR_48,_ although there were no statistical differences. PAR_48 has_ shown better performance in predicting one-month neurological outcomes in patients with PCAS after non-traumatic OHCA than using PCT_48_ alone.

### 4.3. Limitation

This study has several potential limitations. First, it was a retrospective study which may have some potential biases. Second, the sample size was small and the study was conducted as a single-center study. Therefore, our results may not be representative of the overall population. Third, unlike the majority of previous studies, we did not differentiate between therapeutic normothermia and therapeutic hypothermia groups based on the previous studies reporting no association between temperature and prognosis [43]. This will create some limitations in comparison with other previous studies. Fourth, our study did not include patients who did not receive TTM, which may limit the applicability of our findings to patients without TTM. Additionally, this study has evaluated only CPC scores despite that the Core Outcome Set of Cardiac Arrest (COSCA) guideline recommends other tools of neurologic outcome evaluation, such as structured CPC (assessment by semi-structured interview), CPC-Extended, Glasgow Outcome Scale-Extended, and modified Rankin Scale (mRS). Various studies [8,11,14] on the outcome of post-cardiac arrest syndrome are commonly using the CPC score as an evaluation tool for neurological outcomes; hence, we believe that utilization of the same scale is more appropriate to make comparisons. However, we expect further studies to be conducted based on COSCA guidelines to predict neurological outcomes. Finally, since this study evaluated only a one-month prognosis, there are limitations in applying the results to relatively long-term prognosis. We believe further prospective multi-center studies with a longer study period should be conducted to complement our results and to confirm the potential benefits of using PAR as a prognostic factor in patients with PCAS.

## 5. Conclusions

Our findings suggest that PAR_48_ is more effective in predicting one-month neurological outcome than PCT_48_ in patients with PCAS after OHCA.

## Figures and Tables

**Table 1:** Baseline characteristics of study populations.

Variables	1m-mortality			1m-CPC		
	Survivor (n = 131)	Non-Survivor (n = 56)	*p* Value	Good (n = 70)	Poor (n = 117)	*p* Value
Sex, male (%)	98 (52.4)	37 (19.8)	0.285	49 (26.2)	86 (46.0)	0.617
Age (years)	55 (44–64)	58 (47–75)	0.058	56 (44–63)	57 (47–69)	0.361
Location of CA, Residence (%)	66 (94.2)	29 (51.8)	0.001	34 (48.6)	61 (52.1)	0.517
Bystander CPR (%)	121 (92.4)	45 (80.4)	<0.023	64 (91.4)	102 (87.2)	0.476
Shockable rhythm (%)	67 (51.1)	8 (14.3)	<0.001	44 (62.9)	31 (26.5)	<0.001
Etiology of CA (%)			<0.001			<0.001
Cardiac origin	67 (51.1)	10 (17.6)		45 (64.3)	32 (27.4)	
Asphyxia	10 (7.6)	8 (14.3)		5 (7.1)	13 (11.1)	
Other medical condition	41 (31.3)	27 (48.2)		16 (22.9)	52 (44.4)	
Unknown	13 (9.9)	11 (19.6)		4 (5.7)	20 (17.1)	
Collapse to ROSC (min)	24 (16–33)	32 (26–45.70)	<0.001	20 (12.75–29)	30 (22–42.50)	<0.001
Initial pH	7.18 (7.03–7.28)	6.96 (6.83–7.16)	<0.001	7.21 (7.09–7.30)	7.05 (6.90–7.19)	<0.001
Lactate at BL (mmol/L)	7.60 (5.10-9.90)	11.45 (8.02–13.45)	<0.001	7.60 (5.07–9.95)	8.70 (6.30–12.10)	0.031

Values are expressed as number (percentage) and median (interquartile range) as appropriate. CPC, cerebral performance category (good outcome: CPC 1 to 2, poor outcome: CPC 3–5); CA, cardiac arrest; ROSC, return of spontaneous circulation; BL, basal level.

**Table 2:** Comparison of PCT, ALB and PAR according to 1m-mortality and 1m-neurological outcome.

Variables	1m-mortality			1m-CPC		
	Survivor (n = 131)	Non-Survivor (n = 56)	*p* Value	Good (n = 70)	Poor (n = 117)	*p* Value
PCT_0_ (ng/mL)	0.05 (0.05–0.13)	0.09 (0.05–0.39)	0.006	0.05 (0.05–0.11)	0.06 (0.05–0.22)	<0.001
ALB_0_ (g/dL)	3.80 (3.50–4.10)	3.45 (3.12–3.80)	<0.001	3.80 (3.50–4.20)	3.60 (3.20–3.95)	0.003
PAR_0_	0.02 (0.01–0.03)	0.03 (0.02–0.14)	<0.001	0.01 (0.01–0.03)	0.02 (0.01–0.07)	<0.001
PCT_24_ (ng/mL)	1.65 (0.27–6.16)	6.63 (1.48–31.49)	<0.001	0.64 (0.13–2.59)	5.17 (1.79–18.59)	<0.001
ALB_24_ (g/dL)	3.50 (3.20–3.85)	3.15 (2.72–3.58)	<0.001	3.60 (3.30–3.80)	3.30 (2.90–3.60)	0.001
PAR_24_	0.48 (0.08–1.87)	2.62 (0.40–10.52)	<0.001	0.18 (0.04–0.88)	1.63 (0.41–6.00)	<0.001
PCT_48_ (ng/mL)	1.70 (0.29–8.38)	14.98 (1.64–38.57)	<0.001	0.53 (0.17–3.07)	6.75 (1.15–27.38)	<0.001
ALB_48_ (g/dL)	3.30 (2.90-3.60)	2.80 (2.45-3.20)	<0.001	3.45 (3.00–3.70)	3.10 (2.68–3.30)	<0.001
PAR_48_	0.46 (0.09-2.75)	4.88 (0.49-15.15)	<0.001	0.15 (0.04–0.93)	2.23 (0.35–10.53)	<0.001

Values are expressed as number (percentage) and median (interquartile range) as appropriate. CPC, cerebral performance category (good outcome: CPC 1 to 2, poor outcome: CPC 3–5); PCT, procalcitonin; ALB, albumin; PAR, procalcitonin to albumin ratio.

**Table 3:** Univariable and multivariable logistic regression analysis for prediction of 1m-mortality.

Variable	1m-mortality			
	Unadjusted OR (95% CI)	*p* Value	Adjusted OR (95% CI)	*p* Value
PCT_0_	1.058 (0.984–1.138)	0.128	1.064 (0.981–1.154)	0.133
PCT_24_	1.018 (1.005–1.031)	0.006	1.012 (0.999–1.025)	0.068
PCT_48_	1.010 (1.002–1.019)	0.018	1.003 (0.992–1.013)	0.630
PAR_0_	1.239 (0.923–1.663)	0.154	1.249 (0.902–1.729)	0.181
PAR_24_	1.057 (1.018–1.098)	0.004	1.036 (0.997–1.077)	0.067
PAR_48_	1.031 (1.007–1.056)	0.012	1.006 (0.979–1.033)	0.675
Lactate_0_	1.270 (1.150–1.402)	<0.001	1.248 (1.121–1.390)	<0.001
pH_0_	0.008 (0.001–0.059)	<0.001	0.020 (0.002–0.181)	<0.001

Each variable was individually entered into the final model and analyzed separately. OR, odds ratio; CI, confidence interval; PCT, procalcitonin; PAR, procalcitonin to albumin ratio.

**Table 4:** Univariable and multivariable logistic regression analysis for prediction of 1m-CPC.

Variable	1m-CPC			
	Unadjusted OR (95% CI)	*p* Value	Adjusted OR (95% CI)	*p* Value
PCT_0_	1.164 (0.954–1.421)	0.135	1.150 (0.950–1.393)	0.152
PCT_24_	1.080 (1.031–1.131)	0.001	1.055 (1.010–1.103)	0.016
PCT_48_	1.028 (1.006–1.052)	0.014	1.019 (1.001–1.037)	0.034
PAR_0_	1.677 (0.854–3.292)	0.133	1.601 (0.855–2.999)	0.142
PAR_24_	1.254 (1.090–1.442)	0.002	1.167 (1.023–1.330)	0.021
PAR_48_	1.104 (1.028–1.187)	0.007	1.077 (1.012–1.146)	0.020
Lactate_0_	1.098 (1.010–1.194)	0.028	1.042 (0.946–1.149)	0.402
pH_0_	0.015 (0.002–0.098)	<0.001	0.056 (0.007–0.475)	0.008

Each variable was individually entered into the final model and analyzed separately. OR, odds ratio; CI, confidence interval; PCT, procalcitonin; PAR, procalcitonin to albumin ratio.

**Table 5:** ROC analysis of PCT and PAR for prediction of 1m-mortality.

Value	AUC	95% CI	COV	Sensitivity	Specificity	*p*-Value
PCT_0_	0.619	0.545–0.689	0.1	48.2	73.3	0.005
PCT_24_	0.682	0.609–0.749	5.09	55.4	72.8	<0.001
PCT_48_	0.703	0.627–0.772	9.69	53.3	78.3	<0.001
PAR_0_	0.665	0.593–0.733	0.013	91.1	35.9	0.001
PAR_24_	0.684	0.611–0.751	1.912	53.7	76.0	<0.001
PAR_48_	0.710	0.635–0.778	8.629	44.4	88.3	<0.001
Lactate_0_	0.727	0.640–0.808	10.1	62.5	77.86	<0.001
pH_0_	0.723	0.637–0.809	6.97	51.79	86.26	<0.001

Cut-off values were determined by the Youden Index calculated from the ROC curves. DeLong test, PCT_0_ vs. PAR_0_: *p* = 0.009; PCT_24_ vs. PAR_24_: *p* = 0.587; PCT_48_ vs. PAR_48_: *p* = 0.052; Lactate_0_ vs. PCT_0~48_: *p* = 0.071, 0.419, 0.441; Lactate_0_ vs. PAR_0~48_: *p* = 0.297, 0.442, 0.523; pH_0_ vs. PCT_0~48_: *p* = 0.104, 0.512, 0.464; pH_0_ vs. PAR_0~48_: *p* = 0.345, 0.544, 0.545, respectively. AUC, area under the curve; CI, confidence interval; COV, cut-off value; PCT, procalcitonin; PAR, procalcitonin to albumin ratio.

**Table 6:** ROC analysis of PCT and PAR for prediction of 1m-CPC.

Value	AUC	95% CI	COV	Sensitivity	Specificity	*p*-Value
PCT_0_	0.612	0.533–0.677	0.06	49.6	70.0	0.005
PCT_24_	0.787	0.703–0.830	1.65	78.9	72.5	<0.001
PCT_48_	0.763	0.673–0.811	2.0	68.9	72.6	<0.001
PAR_0_	0.647	0.572–0.714	0.015	62.4	62.9	<0.001
PAR_24_	0.790	0.706–0.833	0.5	75.0	73.9	<0.001
PAR_48_	0.772	0.682–0.818	0.275	80.6	62.9	<0.001
Lactate_0_	0.594	0.511–0.678	10.1	41.03	77.14	<0.027
pH_0_	0.700	0.625–0.776	7.07	56.41	80.00	<0.001

Cut-off values were determined by the Youden Index calculated from the ROC curves. DeLong test, PCT_0_ vs. PAR_0_: *p* = 0.039, PCT_24_ vs. PAR_24_: *p* = 0.494, PCT_48_ vs. PAR_48_: *p* = 0.010; Lactate_0_ vs. PCT_0~48_: *p* = 0.833, <0.001, 0.005; Lactate_0_ vs. PAR_0–48_: *p* = 0.405, <0.001, 0.003, respectively. pH_0_ vs. PCT_0~48_: *p* = 0.081, 0.197, 0.477; pH_0_ vs. PAR_0–48_: *p* = 0.310, 0.177, 0.375, respectively AUC, area under the curve; CI, confidence interval; COV, cut-off value; PCT, procalcitonin; PAR, procalcitonin to ALB ratio.

## Data Availability

The datasets generated and analyzed during the current study are not publicly available since they contain potentially identificatory information for each patient; however, they are available from the corresponding author upon reasonable request.
